# PERCEIVED CONTROL, FUNCTION, AND QUALITY OF LIFE AMONG CHRONIC STROKE SURVIVORS: THE MEDIATING EFFECT OF DEPRESSION

**DOI:** 10.1093/geroni/igad104.2444

**Published:** 2023-12-21

## Abstract

This study examined the association between perceived control and function, quality of life (QoL) among Chinese chronic stroke survivors, and explored the mediating effect of depressive symptoms on the relationship between perceived control and ADL, QoL. We conducted a cross-sectional study and assessed depressive symptoms (by the Center for Epidemiological Studies Depression Scale, CES-D), perceived control (by the Perceived Control in Health Care Questionnaire), function (by the Barthel Index, BI), and QoL (by the Short Version of the Stroke Specific Quality of Life Scale, SV-SS-QoL) of 245 stroke survivors at 6 months after onset from three stroke centers in Shanghai, Linyi, and Nanjing from June to December 2021. Model-based causal mediation analysis was used to analyze the mediating effect. Multiple linear regressions showed that higher level of perceived control was significantly associated with better function and QoL (β = 0.22, p< 0.001; β = 0.22, p< 0.001, respectively), and depression symptoms partially mediated the effect of perceived control on function and QoL (β = 0.13, p< 0.001; β = 0.15, p< 0.001, respectively), accounting for 36.07% and 40.60% of the total effect (β = 0.35, p < 0.001, β = 0.37, p < 0.001, respectively). Our study indicates that depressive symptoms shape the association between perceived control and function and QoLamong chronic stroke survivors. Intervention focusing on depressive symptoms may have the potential to improve function and QoL of chronic stroke survivors.

